# Correlations between gastrointestinal and oral microbiota in children with cerebral palsy and epilepsy

**DOI:** 10.3389/fped.2022.988601

**Published:** 2022-11-04

**Authors:** Congfu Huang, Chunuo Chu, Yuanping Peng, Nong Zhang, Zhenyu Yang, Jia You, Fengxiang Wei

**Affiliations:** ^1^ Department of Pediatrics, Longgang District Maternity & Child Healthcare Hospital, Shenzhen, China; ^2^ Shenzhen Middle School, Shenzhen, China; ^3^ The Outpatient Department, Longgang District Social Welfare Center, Shenzhen, China; ^4^ School of Statistics and Data Science, NanKai University, Tianjin, China

**Keywords:** cerebral palsy, epilepsy, oral microbiota, gut microbiota, malnutrition

## Abstract

We here studied the correlation between gut and oral microbiota in children with cerebral palsy and Epilepsy (CPE). We enrolled 27 children with this condition from the social welfare center of Longgang District, collected their oral plaque and stool samples, and analyzed their gut microbiota (GM) and oral microbiota (OM) through 16S rRNA gene sequencing. Taxonomical annotation revealed that the levels of Firmicutes and Bacteroides in the oral cavity were significantly lower in CPE children than in healthy children, whereas the abundance of Actinomycetes increased significantly in CPE children. In addition, *Prevotella*, *Fusobacterium*, and *Neisseria* were the top three abundant genera, representing 15.49%, 9.34%, and 7.68% of the OM and suggesting potential correlations with caries, periodontitis, and malnutrition. For the GM, *Bifidobacterium*, *Bacteroides*, and *Prevotella* were the top three abundant genera in CPE children and probably contributed to the development of chronic inflammation and malnutrition. Furthermore, the OM and GM correlated with each other closely, and the bacterial components of these microbiota in CPE children were remarkably different from those in healthy children, such as *Bifidobacterium*, *Fusobacterium*, *Bacteroides*, and *Neisseria*. Conclusively, dysbiotic OM can translocate to the intestinal tract and induce GM dysbiosis, suggesting the consistency between OM and GM variations. Altered oral and gut microbial structures have potential impacts on the occurrence of clinical diseases such as periodontitis, caries, and malnutrition.

## Introduction

Gut and oral microbiota are the two most abundant microcommunities inhabiting humans, and their impacts on human health are significant. Oral microbiota (OM) are translocated to the gut *via* sputum swallowing (1), and each adult can swallow 600 ml sputum each day, which contains 10^9^/ml bacterial cells. Similarly, 45% of gut microbiota (GM) are also found among the oral microbiota (OM) (1). As previously reported, the levels of periodontitis-associated *Porphyromonas* and *Fusobacterium nucleatum* increased in the GM of liver cirrhosis and colorectal cancer patients, and these bacteria regulated the expression of specific proteins that promote the incidence of these medical conditions (2). In addition, periodontitis and dysbiosis of OM are commonly found to be correlated with inflammatory bowel disease (IBD) (3), and gingival microbiota in liver cirrhosis differed from typical periodontitis-associated microbes (4).

Children with cerebral palsy and epilepsy (CPE) usually have oral health problems, such as periodontitis and dental caries (5, 6). Additionally, increased *F. nucleatum* and *Porphyromonas gingivalis* (7, 8) in the oral cavity can induce GM dysbiosis, thereby causing gastrointestinal dysfunctions such as constipation (9–11). Clinical studies have observed higher incidence of GM dysbiosis, dental caries, and periodontitis in CPE children than in healthy children, partly owing to a decreased level of specific microbes that improve iron, zinc, calcium, and vitamin D synthesis as well as amino acid absorption to prevent dental caries development (12). Our previous report documented the association of disordered GM in CPE children (13) with malnutrition (14). Moreover, the components of GM in CPE children were affected by diets, and gastrointestinal dysfunction was prevalent in children consuming liquid food (15). Although previous studies have explored the impact of OM and GM on human health, the association between OM and GM as well as the combinational effect of the OM and GM on host health require further investigation.

This study recruited 27 children with CPE to analyze oral and gut microbial structures. Clinical phenotypes were also included to understand the correlation of the OM and GM with periodontitis, dental caries, and malnutrition.

## Materials and methods

### Study design and subject enrollment

We here enrolled 27 CPE children (age: 4–14 years) who received liquid food from the social welfare center of Longgang District. The recruited children had no inherited metabolic diseases, gastrointestinal dysplasia, long-term parenteral nutrition, and antibiotic or probiotic exposure 4 weeks before enrollment. Guardians of the enrolled children provided informed consent. Of the 27 children, children had malnutrition, 26 (96.3%) had periodontitis, 22 (81.48%) had dental caries, and 11 (40.74%) had intractable constipation (Supplementary Table S1).

### Sample collection

Oral sampling was performed for children who had not consumed food for at least 2 h. After gargling with sterile saline, two dentists from our hospital collected subgingival microbial samples for the first deciduous or permanent molar at the upper right and stored the samples in buffer (MoBio). Regarding the feces sample, enema was applied if children had not defecated, and then, a caregiver used sterile swabs to collect feces (approximately 5 g). All microbial samples were stored at −80°C within 30 min after sampling.

### DNA extraction and sequencing

The bacterial DNA was extracted from the stool and oral samples by using the PowerSoil® DNA Isolation Kit (Mo Bio Laboratories, Carlsbad, USA) and was analyzed through 1% gel electrophoresis. The eligible DNA was amplified with 16S rRNA V3‒V4 primers (341-FR: CCTACGGGRBGCASCAG, 806-RR: GGACTACNNGGGTATCTAAT) by using the TruSeq DNA PCR-Free kit (Illumina, San Diego, CA, USA). The qualified amplicon library was prepared for high-throughput sequencing with 250-bp paired-end reads using the Miseq platform (Illumina, USA).

### Data processing and bioinformatics analysis

The sequenced raw data were filtered using trimmomatic (v0.39) with parameters (LEADING:3 TRAILING:3 SLIDINGWINDOW:30:15 MINLEN:150). Then, the high-quality paired-end reads were assembled using FLASH software (v1.2.11). USEARCH was applied to cluster the connected sequences to form operational taxonomic units (OTUs). Subsequently, the representative OTUs were taxonomized using the RDP classifier against the Greengenes database (v13.5). Finally, we produced bacterial community composition profiles at the phylum, class, order, family, genus, and species levels.

### Statistical analysis

The principal component analysis method (ade4 package) was applied to the bacterial relative abundance profile at the genus level, and then, the top two principal components were selected to perform a scatter plot and distinguish samples from different groups. Permutational multivariate analysis of variance (PERMANOVA, vegan package) was used to assess the impact of phenotypes on the microbiota. Significant statistical differences were selected when *P* was below 0.05. The Pearson's correlation method was applied to analyze the associations between OM and clinical indicators, GM and clinical indicators, and OM and GM. The absolute value of correlation *P* < 0.05 was considered as a significant correlation. All the data analysis and visualization in this study were conducted using R software (v3.3.3) and the ggplot2 package.

## Results

### Characteristics of OM and GM in CPE children at the phylum level

The top five phyla in the GM were Firmicutes (averaged 32.689%), Bacteroidetes (28.869%), Actinobacteria (27.547%), Proteobacteria (4.698%), and Fusobacteria (1.903%) (Table 1). On the other hand, the abundance of Bacteroidetes (27.808%), Proteobacteria (23.655%), Firmicutes (15.681%), Actinobacteria (13.186%), and Fusobacteria (10.905%) was the highest in the OM (Table 2).

**Table 1:** Top five abundant phyla in oral and gut microbiota.

Gut microbiota			Oral microbiota		
Phylum	Mean (%)	SD (%)	Phylum	Mean (%)	SD (%)
Firmicutes	32.689	13.083	Bacteroidetes	27.808	9.716
Bacteroidetes	28.869	20.77	Proteobacteria	23.655	20.185
Actinobacteria	27.547	19.026	Firmicutes	15.681	10.388
Proteobacteria	4.698	3.689	Actinobacteria	13.186	11.459
Fusobacteria	1.903	2.422	Fusobacteria	10.905	8.052

**Table 2:** Top 15 abundant genera in oral and gut microbiota.

Gut microbiota			Oral microbiota		
Genera	Average (%)	SD (%)	Genera	Average (%)	SD (%)
*Bifidobacterium*	21.73	16.507	*Prevotella*	15.49	12.253
*Bacteroides*	9.991	10.265	*Fusobacterium*	9.34	7.831
*Prevotella*	8.718	14.52	*Neisseria*	7.68	11.812
*Parabacteroides*	4.7	4.989	*Actinomyces*	5.494	5.84
*Collinsella*	4.631	4.213	*Brachymonas*	3.881	6.797
*Megasphaera*	2.992	3.79	*Streptococcus*	3.408	4.339
*Clostridium_IV*	2.633	4.351	*Porphyromonas*	3.082	3.226
*Oscillibacter*	2.317	2.11	*Capnocytophaga*	3.005	4.481
*Lachnospiracea_incertae_sedis*	2.215	2.705	*Corynebacterium*	2.509	4.674
*Streptococcus*	2.09	3.337	*Campylobacter*	2.09	2.005
*Akkermansia*	1.896	2.422	*Veillonella*	1.669	2.424
*Alloprevotella*	1.837	5.099	*Rothia*	1.629	4.206
*Faecalibacterium*	1.802	2.757	*Bifidobacterium*	1.445	3.523
*Megamonas*	1.525	4.538	*Leptotrichia*	1.401	4.078
*Clostridium_XIVa*	1.396	1.055	*Treponema*	1.398	2.831

### Characteristics of OM and GM in CPE children at the genus level

We selected the top 15 genera in the OM and GM to perform a comparative analysis. Bacterial composition is different between the OM and GM (Table 2). *Bifidobacterium*, *Prevotella*, and *Streptococcus* were identified in both OM and GM. The levels of *Bifidobacterium*, *Bacteroidetes*, and *Prevotella* were the highest in the GM, but the top three genera in the OM were *Prevotella*, *Fusobacterium*, and *Neisseria*.

### Relations between the OM/GM and clinical phenotypes

**Figure 1:**
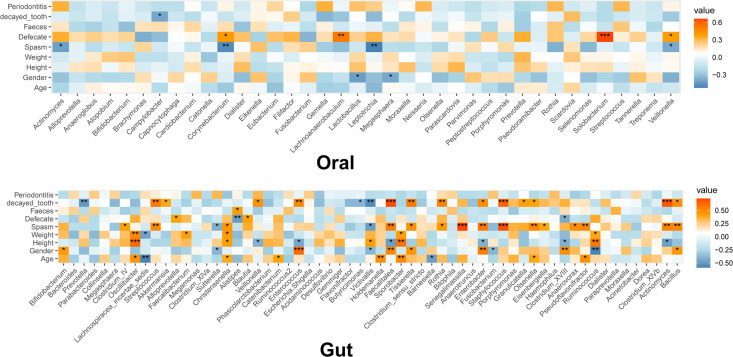
Heatmap for the association of microbiota with clinical phenotypes. The color represents the correlation coefficient: green, negative correlation; red, positive correlation. The asterisk represents the significance of correlation: *, **, ***, and **** represent *P* = 0.05, 0.01, 0.001, and 0.0001, respectively.

**Figure 2:**
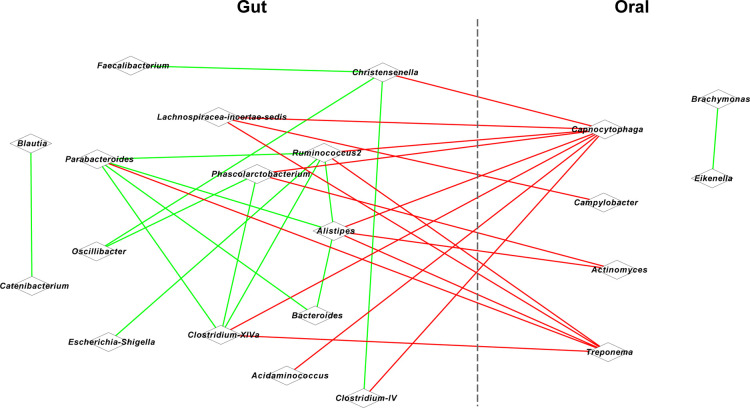
Co-occurrence network for oral and gut microbiota. Red lines represent a positive correlation, and blue lines represent a negative correlation.

We identified a positive correlation between the defecation frequency and the levels of *Solobacterium* (*r* = 0.662, *P* = 0.000), *Lachnoanaerobaculum* (*r* = 0.522, *P* = 0.005), *Corynebacterium* (*r* = 0.408, *P* = 0.035), and *Veillonella* (*r* = 0.390, *P* = 0.044) in the OM (Figure 1). *Actinomyces* (*r* = −0.413, *P* = 0.032), *Corynebacterium* (*r* = −0.492, *P* = 0.009), *Leptotrichia* (*r* = −0.524, *P* = 0.005), and *Veillonella* (*r* = −0.385, *P* = 0.047) in the OM correlated negatively with the spasm frequency (Figure 1). Regarding the GM, the defecation frequency associated positively with *Alloprevotella* (*r* = 0.396, *P* = 0.041) and *Blautia* (*r* = 0.402, *P* = 0.038), but negatively with *Alistipes* (*r* = −0.488, *P* = 0.010) and *Clostridium*_XVIII (*r* = −0.424, *P* = 0.027) (Figure 2). In addition, the spasm frequency was positively associated with *Senegalimassilia* (*r* = 0.724, *P* = 0.000), *Staphylococcus* (*r* = 0.698, *P* = 0.000), *Actinomyces* (*r* = 0.521, *P* = 0.005), and *Bacillus* (*r* = 0.515, *P* = 0.006), but negatively associated with *Sutterella* (*r* = −0.411, *P* = 0.033) and *Victivallis* (*r* = −0.475, *P* = 0.012) (Figure 2). These findings suggest the significant association of defecation and spasm frequencies with the OM and GM; however, the underlying mechanisms should be further explored.

### Association between OM and GM in the CPE children

Given the significant correlation between the defection/spasm frequency and OM/GM, we further conducted association analysis for the OM and GM. Based on Spearman's coefficient (*P* < 0.05), we found that oral *Capnocytophaga* was positively correlated with intestinal *Christensenella* (*r* = 0.847), Alistipes (*r* = 0.850), and *Clostridium*-IV (*r* = 0.837) (Figure 2). A positive correlation was also identified between oral *Campylobacter* and intestinal *Lachnospiracea-incertae-sedis* (*r* = 0.837), between oral *Actinomyces* and intestinal *Phascolarctobacterium* (*r* = 0.842) and *Alistipes* (*r* = 0.834), and between oral *Treponema* and intestinal *Clostridium*-XlVa (*r* = 0.845), *Parabacteroides* (*r* = 0.849), and *Alistipes* (*r* = 0.851).

## Discussion

Microbial components in the OM and GM are in dynamic homeostasis, which is maintained by various factors such as bile acids in the gut lumen (16–18). Although millions of microbial cells can be swallowed from the oral cavity into the gastrointestinal tract, the stomach and small intestine function as barriers for these OM and GM because 99% of swallowed microbes are killed at these two sites. Schmidt et al. (19) observed that microbial strains in the gut are translocated from the oral cavity, and a correlation analysis found the oral–gut transmission of opportunistic pathogenic agents as well as dental caries/periodontitis-associated pathogens. For instance, sputum-derived *Klebsiella* in IBD patients induced intestinal inflammation in gnotobiotic mice, suggesting the role played by microbial transmission in etiology (20). The aforementioned findings implicated the translocation of oral microbes to the gut, which thus can impact the functional network of the GM.

Increasing evidence has demonstrated the association of OM with dental health problems such as periodontitis and dental caries (21–23). Clinical studies have indicated that 86% of children with cerebral palsy (CP) had mild-to-moderate gingivitis (24) and 52.5%–81% had dental caries (25–27). Similarly, we found 96.3% incidence of periodontitis and 81.48% incidence of gingivitis in these children, which can be partly explained by their impaired oral functions, such as occlusion and swallow (25–27). Studies have observed the association of oral health with OM, including *Porphyromonas*, *Treponem*a, *F. nucleatum*, *Pseudoramibacter*, *Streptococcus*, *Prevotella* as well as lactic acid-producing bacteria (7, 8, 28–34). Liu et al. found that the core caries-associated microbiota in CP children included Prevotella, Alloprevotella, Actinomyces, Catonella, and Streptobacillus, while Capnocytophaga and Campylobacter were dental health-associated microbiota in these children (35). In line with other studies, the present study identified the accumulation of *Prevotella*, *Fusobacterium*, *Neisseria*, *Actinomyces*, *Brachymonas*, *Streptococcus*, *Porphyromonas*, *Capnocytophaga*, and *Campylobacter* in the OM of CPE children (7, 8, 26, 27, 35).

Our studies have found that the abundance of Bacteroides, Faecalibacterium, Lactobacillus, Ruminococcus, Roseburia and other beneficial bacteria is significantly reduced in the gut of CP children, whereas the abundance of harmful bacteria such as Streptococcus, Collinsella, Alistipes, Eggerthella, Enterococcus, and Veillonella is significantly increased (13, 15). Neurodegenerative diseases were mainly attributed to Streptococcus (13). Moreover, we found that the abundance of Bifidobacterium was significantly increased in CP children who consumed liquid food, while the abundance of Prevotella increased significantly in CP children who ate normal food (15). Given the strong proof for the association of malnutrition with GM (36), this study also explored GM structures for enrolled children who had malnutrition (40.7% had intractable constipation). As previously reported (13, 15), in this study, we observed high levels of pathogens in the recruited children, including Collinsella, Enterococcus, and Streptococcus, and the levels of beneficial microbes such as Bacteroides, Prevotella, Roseburia, and Lactobacillus were depleted (37, 38). These beneficial microbes can produce short-chain fatty acids that can function as nutrients, protect gut barriers, and alleviate inflammation (39). Therefore, this is the rationale to speculate the role of GM dysbiosis in malnutrition in our study.

In addition, we found that oral disease-associated microbial components are significantly associated with the gut components (7, 8, 25–28, 32–34), which correlated with the protection offered by gut barrier and mitigation of intestinal inflammation (15, 40–42). This suggested the translocation of disordered OM to the gut, which modulate the gut microenvironment and health.

In general, oral microbes are among the sources of GM, and the oral–gut transmission plays a major role in gastrointestinal health (19). As our previous study presented results similar to those of this study, we suspected that disordered OM used various mechanisms to affect gut microenvironments; gut dysfunction can impact gastrointestinal health and disease development. Our findings suggested the potential of probiotics/prebiotics intervention in modulating the OM and GM as well as improving health. However, the lack of healthy children and a small sample size limited the exploration of the association of OM/GM with the health of CPE children. Longitudinal data will also improve the understanding of mechanisms for the correlation between OM/GM and health.

## Data Availability

The dataset generated for this study is accessible from the NCBI sequence Archive (SRA) database with project number PRJNA853735.

## References

[1] Iwauchi M Horigome A Ishikawa K Mikuni A Nakano M Xiao JZ Relationship between oral and gut microbiota in elderly people. Immun Inflamm Dis. (2019) 7(3):229–36. 10.1002/iid3.266 31305026 PMC6688080

[2] Xu T Pei Y Chen L Wu X Geng H Zhou J Oral flora basis of traditional Chinese medicine tongue coating and its relationship with intestinal Flora. J Tradit Chin Med. (2019) 60(3):202–5. 10.13288/j.11-2166/r.2019.03.006

[3] Papageorgiou SN Hagner M Nogueira AV Franke A Jager A Deschner J. Inflammatory bowel disease and oral health: systematic review and a meta-analysis. J Clin Periodontol. (2017) 44(4):382–93. 10.1111/jcpe.12698 28117909

[4] Jensen A Ladegaard Gronkjaer L Holmstrup P Vilstrup H Kilian M. Unique subgingival microbiota associated with periodontitis in cirrhosis patients. Sci Rep. (2018) 8(1):10718. 10.1038/s41598-018-28905-w 30013030 PMC6048062

[5] Bensi C Costacurta M Docimo R. Oral health in children with cerebral palsy: a systematic review and meta-analysis. Spec Care Dentist. (2020) 40(5):401–11. 10.1111/scd.12506 32815638

[6] Akhter R Hassan NM Martin EF Muhit M Haque MR Smithers-Sheedy H Risk factors for dental caries among children with cerebral palsy in a low-resource setting. Dev Med Child Neurol. (2017) 59(5):538–43. 10.1111/dmcn.13359 27935024

[7] Xu Y Jia Y Chen L Huang W Yang D. Metagenomic analysis of oral microbiome in young children aged 6-8 years living in a rural isolated Chinese province. Oral Dis. (2018) 24(6):1115–25. 10.1111/odi.12871 29667264

[8] Yang Z Liu B. Research progress on the microecology of dental plaque in caries. Int J Stomatol. (2020) 47(5):506–14. 10.7518/gjkq.2020032

[9] Engevik MA Danhof HA Ruan W Engevik AC Chang-Graham AL Engevik KA Fusobacterium nucleatum secretes outer membrane vesicles and promotes intestinal inflammation. mBio. (2021) 12(2):e02706–20. 10.1128/mBio.02706-20 33653893 PMC8092269

[10] Kitamoto S Nagao-Kitamoto H Hein R Schmidt TM Kamada N. The bacterial connection between the oral cavity and the gut diseases. J Dent Res. (2020) 99(9):1021–9. 10.1177/0022034520924633 32464078 PMC7375741

[11] Ferreira A Eveloff RJ Freire M Santos M. The impact of oral-gut inflammation in cerebral palsy. Front Immunol. (2021) 12:619262. 10.3389/fimmu.2021.619262 33717115 PMC7953843

[12] Shishniashvili T Suladze T Makhviladze M Kalandaze M Margvelashvili V. Dental diseases and intestinal dysbiosis among children. J Clin Pediatr Dent. (2018) 42(3):217–20. 10.17796/1053-4628-42.3.9 29698134

[13] Huang C Li Y Feng X Li D Li X Ouyang Q Distinct gut microbiota composition and functional category in children with cerebral palsy and epilepsy. Front Pediatr. (2019) 7:394. 10.3389/fped.2019.00394 31646147 PMC6779726

[14] Trivic I Hojsak I. Evaluation and treatment of malnutrition and associated gastrointestinal complications in children with cerebral palsy. Pediatr Gastroenterol Hepatol Nutr. (2019) 22(2):122–31. 10.5223/pghn.2019.22.2.122 30899688 PMC6416384

[15] Huang C Li X Wu L Wu G Wang P Peng Y The effect of different dietary structure on gastrointestinal dysfunction in children with cerebral palsy and epilepsy based on gut microbiota. Brain Dev. (2021) 43(2):192–9. 10.1016/j.braindev.2020.09.013 33071106

[16] Martinsen TC Bergh K Waldum HL. Gastric juice: a barrier against infectious diseases. Basic Clin Pharmacol Toxicol. (2005) 96(2):94–102. 10.1111/j.1742-7843.2005.pto960202.x 15679471

[17] Ridlon JM Kang DJ Hylemon PB Bajaj JS. Bile acids and the gut microbiome. Curr Opin Gastroenterol. (2014) 30(3):332–8. 10.1097/MOG.0000000000000057 24625896 PMC4215539

[18] Imhann F Bonder MJ Vich Vila A Fu J Mujagic Z Vork L Proton pump inhibitors affect the gut microbiome. Gut. (2016) 65(5):740–8. 10.1136/gutjnl-2015-310376 26657899 PMC4853569

[19] Schmidt TS Hayward MR Coelho LP Li SS Costea PI Voigt AY Extensive transmission of microbes along the gastrointestinal tract. eLife. (2019) 8:e42693. 10.7554/eLife.42693 PMC6424576 30747106

[20] Atarashi K Suda W Luo C Kawaguchi T Motoo I Narushima S Ectopic colonization of oral bacteria in the intestine drives TH1 cell induction and inflammation. Science. (2017) 358(6361):359–65. 10.1126/science.aan4526 29051379 PMC5682622

[21] Minty M Canceil T Serino M Burcelin R Terce F Blasco-Baque V. Oral microbiota-induced periodontitis: a new risk factor of metabolic diseases. Rev Endocr Metab Disord. (2019) 20(4):449–59. 10.1007/s11154-019-09526-8 31741266

[22] Wang Y Zhang J Chen X Jiang W Wang S Xu L Profiling of oral microbiota in early childhood caries using single-molecule real-time sequencing. Front Microbiol. (2017) 8:2244. 10.3389/fmicb.2017.02244 29187843 PMC5694851

[23] Qian W Ma T Ye M Li Z Liu Y Hao P. Microbiota in the apical root canal system of tooth with apical periodontitis. BMC Genom. (2019) 20(Suppl 2):189. 10.1186/s12864-019-5474-y PMC6456935 30967114

[24] Diéguez-Pérez M, de Nova-García MJ, Mourelle-Martínez MR, Bartolomé-Villar B. Oral health in children with physical (cerebral palsy) and intellectual (down syndrome) disabilities: systematic review I. *J Clin Exp Dent*. (2016) 8(3):e337–43. 10.4317/jced.52922 PMC4930646 27398187

[25] Ferreira de Camargo MA Frias AC Antunes JL. The incidence of dental caries in children and adolescents who have cerebral palsy and are participating in a dental program in Brazil. Spec Care Dentist. (2011) 31(6):210–5. 10.1111/j.1754-4505.2011.00213.x 22070360

[26] Sinha N Singh B Chhabra KG Patil S. Comparison of oral health status between children with cerebral palsy and normal children in India: a case-control study. J Indian Soc Periodontol. (2015) 19(1):78–82. 10.4103/0972-124X.145800 25810598 PMC4365163

[27] Chen X Hou M Meng L Yu J Wu X. The correlation between dental caries and gross motor function for children with cerebral palsy. Chinese J Rehabilitation Med. (2017) 32(7):829–30. 10.3969/j.issn.1001-1242.2017.07.021

[28] Xu W Zhou W Wang H Liang S. Roles of Porphyromonas gingivalis and its virulence factors in periodontitis. Adv Protein Chem Struct Biol. (2020) 120:45–84. 10.1016/bs.apcsb.2019.12.001 32085888 PMC8204362

[29] Ateia IM Sutthiboonyapan P Kamarajan P Jin T Godovikova V Kapila YL Treponema denticola increases MMP-2 expression and activation in the periodontium via reversible DNA and histone modifications. Cell Microbiol. (2018) 20(4):10.1111/cmi.12815. 10.1111/cmi.12815 29205773 PMC5842108

[30] Lin S Jiao J Wu Y Zhao L. Research progress of correlation between Fusobacterium nucleatum and gastrointestinal diseases. Chinese J Pract Stomatol. (2020) 13(4):237–42. 10.19538/j.kq.2020.04.010

[31] Tatikonda A Sudheep N Biswas KP Gowtham K Pujari S Singh P. Evaluation of bacteriological profile in the apical root segment of the patients with primary apical periodontitis. J Contemp Dent Pract. (2017) 18(1):44–8. 10.5005/jp-journals-10024-1986 28050984

[32] Mohammed SR Anand N Chandrasekaran SC Mahalakshmi K Padmavathy K. Evaluation of periodontal status and detection of dialister pneumosintes in cerebral palsy individuals: a case-control study. Indian J Dent Res. (2018) 29(6):768–72. 10.4103/ijdr.IJDR_582_15 30589006

[33] Luo YX Sun ML Shi PL Liu P Chen YY Peng X. Research progress in the relationship between veillonella and oral diseases. Hua Xi Kou Qiang Yi Xue Za Zhi. (2020) 38(5):576–82. 10.7518/hxkq.2020.05.018 33085245 PMC7573782

[34] Kim BG, Cho AY, Kim SS, Lee SH, Shin HS, Yoon HJ, et al. A case of peritoneal dialysis-associated peritonitis by Rothia mucilaginosa. *Kidney Res Clin Pract*. (2015) 34(3):185–7. 10.1016/j.krcp.2015.02.005 PMC4608873 26484045

[35] Liu M Shi Y Wu K Xie W Ser HL Jiang Q From mouth to brain: distinct supragingival plaque microbiota composition in cerebral palsy children with caries. Front Cell Infect Microbiol. (2022) 12:814473. 10.3389/fcimb.2022.814473 35480234 PMC9037539

[36] Pekmez CT Dragsted LO Brahe LK. Gut microbiota alterations and dietary modulation in childhood malnutrition - the role of short chain fatty acids. Clin Nutr. (2019) 38(2):615–30. 10.1016/j.clnu.2018.02.014 29496274

[37] Subramanian S Huq S Yatsunenko T Haque R Mahfuz M Alam MA Persistent gut microbiota immaturity in malnourished Bangladeshi children. Nature. (2014) 510(7505):417–21. 10.1038/nature13421 24896187 PMC4189846

[38] Ghosh TS Gupta SS Bhattacharya T Yadav D Barik A Chowdhury A Gut microbiomes of Indian children of varying nutritional status. PLoS One. (2014) 9(4):e95547. 10.1371/journal.pone.0095547 24763225 PMC3999041

[39] Fischbach MA Sonnenburg JL. Eating for two: how metabolism establishes interspecies interactions in the gut. Cell Host Microbe. (2011) 10(4):336–47. 10.1016/j.chom.2011.10.002 22018234 PMC3225337

[40] Zhang M Ma W Zhang J He Y Wang J. Analysis of gut microbiota profiles and microbe-disease associations in children with autism spectrum disorders in China. Sci Rep. (2018) 8(1):13981. 10.1038/s41598-018-32219-2 30228282 PMC6143520

[41] Parthasarathy G Chen J Chen X Chia N O'Connor HM Wolf PG Relationship between microbiota of the colonic mucosa vs. feces and symptoms, colonic transit, and methane production in female patients with chronic constipation. Gastroenterology. (2016) 150(2):367–79.e1. 10.1053/j.gastro.2015.10.005 26460205 PMC4727996

[42] Zhu K Zhang Z. The role of Akkermansia muciniphila in the gut microbiota-targeted therapy for gastrointestinal diseases. Chinese J Gastroenterol. (2020) 25(1):43–6. 10.3969/j.issn.1008-7125.2020.01.009

